# Central and Peripheral Sensitization in Temporomandibular Disorders: Proposed Mechanisms of Botulinum Toxin Therapy

**DOI:** 10.3390/toxins18010028

**Published:** 2026-01-06

**Authors:** Basit Ali Chaudhry, Christopher L. Robinson, Edoardo Caronna, Freda Dodd-Glover, Amrittej Singh Virk, Mario Fernando Prieto Peres, Hope L. O’Brien, Marcela Romero-Reyes, Sait Ashina

**Affiliations:** 1 Department of Neurology, Danish Headache Center, Copenhagen University Hospital—Rigshospitalet, 2100 Copenhagen, Denmark; 2 Department of Clinical Medicine, Faculty of Health and Medical Sciences, University of Copenhagen, 2200 Copenhagen, Denmark; 3 Department of Anesthesiology and Critical Care Medicine, The John Hopkins University School of Medicine, Baltimore, MD 21205, USA; 4 Headache and Neurological Pain Research Group, Vall d’Hebron Institut de Recerca (VHIR), Department of Medicine, Universitat Autònoma de Barcelona, 08035 Barcelona, Spain; 5 Headache Unit, Neurology Department, Vall d’Hebron University Hospital, 08035 Barcelona, Spain; 6 Department of Medicine and Therapeutics, Korle Bu Teaching Hospital, Accra P.O. Box 77, Ghana; 7 Texas TMJ Sleep & Facial Pain, Southlake, TX 76092, USA; 8 Instituto de Psiquiatria, HCFMUSP, Faculdade de Medicina da Universidade de São Paulo, São Paulo 05403-903, Brazil; 9 Department of Neurology, Hospital Albert Einstein, São Paulo 05403-903, Brazil; 10 Headache Center of Hope, Cincinnati, OH 45236, USA; 11 Department of Pain and Neural Sciences, Brotman Facial Pain Clinic, University of Maryland School of Dentistry, Baltimore, MD 21201, USA; 12 Department of Neurology, BIDMC Comprehensive Headache Center, Harvard Medical School, Beth Israel Deaconess Medical Center, Boston, MA 02215, USA; 13 Department of Anesthesia, Critical Care and Pain Medicine, BIDMC Comprehensive Headache Center, Harvard Medical School, Beth Israel Deaconess Medical Center, Boston, MA 02215, USA

**Keywords:** allodynia, central sensitization, BoNT-A, botulinum toxin therapy, temporomandibular disorders, TMD, trigeminal nociceptive system

## Abstract

Temporomandibular disorders (TMDs) are common musculoskeletal chronic orofacial pain conditions involving peripheral and central sensitization within trigeminal nociceptive pathways, manifesting as mechanical allodynia and functional impairment. Botulinum toxin type A (BoNT-A) has been explored as a treatment targeting both muscle hyperactivity and nociceptive modulation. Preclinical and clinical evidence demonstrate that BoNT-A reduces peripheral neurotransmitter release, neurogenic inflammation, and central neuronal excitability, leading to attenuation of mechanical allodynia in TMD models and patients. Clinical trials show modest and variable analgesic effects, with patients displaying sensory sensitization appearing to respond more favorably, though methodological heterogeneity limits definitive conclusions. Safety concerns related to muscle weakening, changes in bone density, and structural changes underscore the need for standardized protocols optimizing dosing and monitoring, in addition to prospective studies. These findings suggest that BoNT-A may serve as an adjunctive, mechanism-based therapy within multimodal TMD management. Future research should focus on standardized sensory phenotyping and trial design to clarify BoNT-A’s role in modulating central sensitization and improving patient outcomes.

## 1. Introduction

Temporomandibular disorders (TMD) encompass a group of musculoskeletal conditions affecting the temporomandibular joint (TMJ), muscles of mastication, and associated structures [1,2,3]. They represent one of the most common causes of chronic orofacial pain, with lifetime prevalence estimated at 25–35% [4,5,6]. Although the majority of TMDs are acute and temporary, tending toward remission or improvement in symptoms, a substantial subset may develop persistent pain and dysfunction, causing psychosocial distress affecting quality of life [7]. TMDs are complex and multifactorial, and evidence has shown that the establishment of the cycle of persistent pain involves different biopsychosocial factors in addition to comorbidities particularly overlapping pain conditions, such as primary headache disorders (including migraine and medication overuse headache), anxiety and depression, irritable bowel syndrome (IBS), fibromyalgia, lower back pain, sleep disorders, vulvodynia, and chronic fatigue, together influencing pain amplification [8,9].

The most common painful TMDs, as defined by current DC/TMD criteria, include arthralgia, myalgia, myofascial pain, and headache attributed to TMD [10]. Patients often present with more than one diagnosis. However, myogenous disorders, particularly myalgia and myofascial pain, remain the most prevalent [10,11,12]. It is important to mention that patients may present more than one TMD diagnosis, but from all TMDs, the most common are of masticatory muscle origin, such as myalgia and myofascial pain [11,12].

Chronic temporomandibular disorders share neurobiological features with primary headache disorders, particularly migraine. Furthermore, studies show that migraine is the most common primary headache disorder with a prevalence of 40% in TMD patients [13]. Both involve abnormal trigeminal nociceptive processing characterized by cutaneous allodynia, lowered pain thresholds, and impaired descending inhibition, all markers of central sensitization [2]. Importantly, not all TMD cases exhibit cutaneous allodynia. In chronic TMD, central sensitization may develop, manifested clinically as allodynia and hyperalgesia upon examination. Peripheral sensitization, such as that arising from TMJ injury, could evolve into central sensitization over time. Myofascial pain provides the clearest example of cutaneous allodynia, followed by TMJ arthralgia in selected cases [14,15]. Inflammation of the masseter muscle may contribute to intracranial nociceptive signaling and serve as a priming mechanism for central sensitization. In preclinical studies, inflammation of the masseter as a surrogate of masticatory TMD mediated intracranial nociception and priming [16]. Functional imaging demonstrated overlapping alterations in thalamic, insular, and brainstem pain networks, suggesting a shared pathophysiological substrate [17,18]. In chronic TMD, sensitization manifests as pain spread beyond the orofacial region and heightened excitability within trigeminal and extra-trigeminal pathways [15,19,20,21,22,23,24,25]. These features correlate with a higher pain intensity, greater disability, and reduced response to conventional therapies [26,27], underscoring the need to address both peripheral and central pain mechanisms.

TMD management is recommended to be conservative and reversible [3,28]. This involves an integrated patient-centered plan tailored to the diagnosis and patient needs, combining non-pharmacological and pharmacological modalities. This includes education and self-management, physical therapy, behavioral therapy, intraoral orthotics (such as stabilization appliances, trigger point injection therapy, and simple analgesics), anti-inflammatories, and muscle relaxants. Sometimes, it may include the use of antidepressants such as tricyclics [3,28]. Yet, in many patients, pain persists despite adequate conservative therapy. In some individuals, this reflects central sensitization, while in others, persistent pain may relate to an underlying structural abnormality. Refractory cases, therefore, highlight the need for treatments that can target both peripheral pathology and central pain mechanisms.

Botulinum toxin type A (BoNT-A) has been used in TMD and sleep bruxism to reduce masticatory muscle hyperactivity and local myofascial pain [29,30,31]. The evidence indicates broader mechanisms, including inhibition of neurotransmitters such as glutamate and substance P, modulation of transient receptor potential channels, and attenuation of peripheral sensitization via C fiber inhibition [32,33]. Additional findings suggest retrograde axonal transport and possible modulation of nociceptive signaling within the trigeminal nucleus caudalis (TNC) and higher brain regions [34,35,36]. These data provide a biological rationale for targeting both peripheral and central sensitization in TMD.

Despite this mechanistic rationale, clinical findings remain inconsistent. Differences in study design, dosing, injection mapping, and outcome measures, together with insufficient characterization of central sensitization, have limited comparability across trials. This review critically appraises current evidence linking central sensitization and allodynia to TMD pathophysiology and evaluates how BoNT-A may modulate these mechanisms at both peripheral and central levels. By focusing on the interface between neurobiology and clinical efficacy, we aim to clarify whether BoNT-A’s analgesic effects in TMD extend beyond muscle relaxation to genuine desensitization of the trigeminal system. Unlike existing systematic and umbrella reviews that primarily evaluate clinical efficacy, this narrative review focuses on the mechanistic and pathophysiological basis underlying heterogeneous clinical responses to BoNT-A in TMD, integrating evidence across experimental, translational, and clinical domains.

## 2. Search Strategy and Review Approach

This article is a narrative review synthesizing experimental, clinical, and translational evidence on TMD, central sensitization, and BoNT-A mechanisms. We searched PubMed for “temporomandibular disorder” or “TMD” in combination with the terms “myalgia”, “myofascial pain”, “jaw muscle pain”, “arthralgia”, “central sensitization”, “cutaneous allodynia”, “hyperalgesia”, “trigeminal system”, “trigeminal nociception”, “trigeminal nucleus caudalis”, “peripheral sensitization”, “masseter inflammation”, “botulinum toxin”, “BoNT-A”, and “onabotulinumtoxinA”. We also searched for clinical trials by combining “TMD” or “temporomandibular disorder” with “botulinum toxin”, “BoNT-A”, “onabotulinumtoxinA”, and “randomized controlled trial”. Additional relevant articles were identified by reviewing the reference lists of key publications. As this is a narrative review, no formal systematic methodology was applied, and studies were selected based on relevance to the mechanistic and clinical questions addressed. The literature search was conducted and last updated in November 2025.

## 3. Anatomy and Pathophysiology of TMD Pain

### Anatomical Consideration

**Figure 1:**
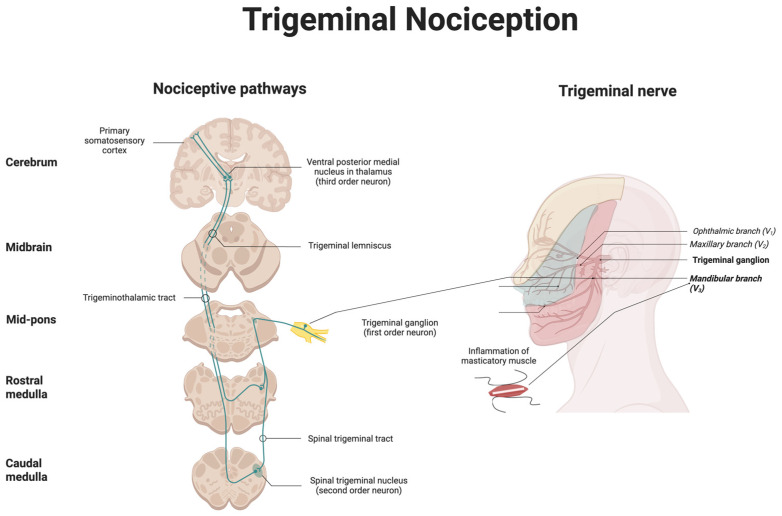
Trigeminal nociceptive pathway and peripheral innervation relevant to TMD. Schematic overview of the trigeminal pain pathway, highlighting nociceptive processing relevant to temporomandibular disorders (TMD). First-order neurons in the trigeminal ganglion transmit nociceptive input primarily from the temporomandibular joint (TMJ) and masticatory muscles via the mandibular branch (V_3_), which mediates pain in both arthrogenous and myogenous forms of TMD. To a lesser extent, afferents from adjacent orofacial tissues project through the ophthalmic (V_1_) and maxillary (V_2_) branches. These fibers converge on second-order neurons in the spinal trigeminal nucleus (TNC) within the caudal medulla, where integration of craniofacial nociceptive input occurs. Ascending axons travel through the trigeminothalamic tract to third-order neurons in the ventroposteromedial (VPM) thalamic nucleus, and finally to cortical regions such as the primary somatosensory, insular, and anterior cingulate cortices, where higher-order processing and sensitization take place. Emphasis on the mandibular branch (V_3_), TMJ, and masticatory muscles distinguishes TMD-related nociception from other orofacial pain syndromes [43]. This figure was created using BioRender (BioRender.com, Toronto, Canada; web-based platform, accessed November 2025).

Temporomandibular disorders develop within a complex anatomical and neurofunctional system integrating joint structures, masticatory muscles, and the trigeminal pathway [1]. The TMJ is a synovial articulation between the mandibular condyle and the temporal bone, separated by a fibrocartilaginous disk that permits both rotational and translational movement during mastication and speech [1]. These motions are coordinated by the masseter, temporalis, and medial and lateral pterygoid muscles under precise motor control from the mandibular branch (V3) of the trigeminal nerve. The same division provides dense sensory innervation to the TMJ capsule, ligaments, periarticular tissues, and orofacial skin, forming an extensive afferent network rich in nociceptors and mechanoreceptors [1]. This organization enables fine motor coordination, creating a substrate through which mechanical overload, microtrauma, or inflammation can activate peripheral nociceptors and drive convergent input to second-order neurons in the TNC, an early step in the development of temporomandibular disorders, pain, and sensitization of these neurons [20,37,38]. Ascending projections from the TNC relay through third-order neurons in the thalamus, particularly the ventroposteromedial nucleus (VPM), to somatosensory, insular, and anterior cingulate cortices, where further amplification and maladaptive plasticity can occur [39]. Central sensitization in chronic TMD is characterized by increased activation of the somatosensory cortex, prefrontal cortex, and anterior cingulate in addition to reduced thalamic activation [18,40,41]. Anatomical convergence between masticatory nociceptive inputs can engage trigeminal pathways implicated in migraine and other cephalalgias, predisposing TMD to central sensitization [16,42] (see Figure 1).

## 4. Peripheral and Central Mechanisms

**Figure 2:**
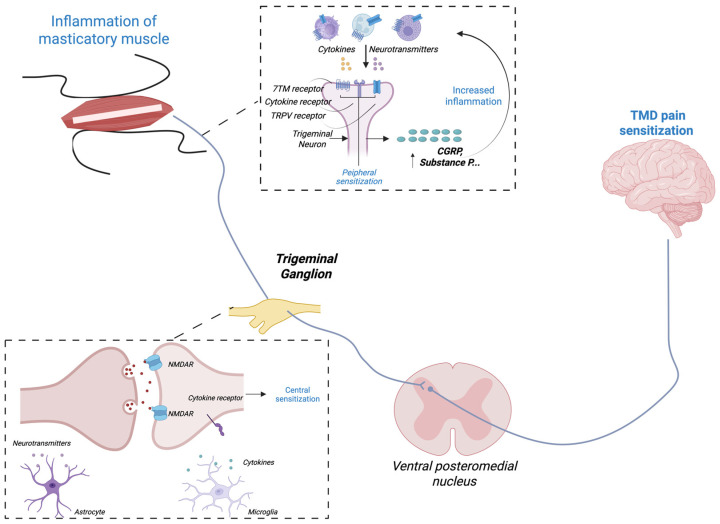
Trigeminal pathways with peripheral and central sensitization nodes. Schematic illustration of peripheral and central sensitization mechanisms contributing to pain in temporomandibular disorders (TMDs). Inflammatory activity of the masticatory muscles activates trigeminal afferents through receptors such as TRPV and cytokine receptors, promoting peripheral sensitization and release of neuropeptides, notably calcitonin gene-related peptide (CGRP) and substance P. CGRP plays a key role in neurogenic inflammation and amplification of trigeminal nociceptive signaling, as demonstrated in experimental models of TMD [49]. Persistent nociceptive input from the masticatory muscle enhances excitatory neurotransmission and cytokine signaling within the trigeminal ganglion and central nuclei, facilitating central sensitization and ultimately TMD-related pain hypersensitivity. NMDAR: N-methyl-D-aspartate receptor; CGRP: calcitonin gene-related peptide. This figure was created using BioRender. Persistent nociceptive input from the periphery activates second-order neurons in the TNC [55]. Afferents from the TMJ, masticatory muscles, and orofacial skin converge within the TNC, creating a substrate for central sensitization [55]. Repetitive C-fiber stimulation induces sustained increases in neuronal firing through N-methyl-D-aspartate (NMDA) receptor activation, calcium influx, and downstream kinase signaling, which strengthen synaptic efficacy and expand receptive fields [55]. These changes mirror mechanisms described in migraine and other centrally sensitized pain states, underscoring shared pathophysiological principles [16,56]. These changes result in pain amplification, hyperalgesia, and spread of pain beyond the original site of injury [53]. Disinhibition further reinforces this process. Reduced GABAergic and glycinergic tone, combined with impaired descending inhibitory control from supraspinal centers, increases the excitability of trigeminal nociceptive neurons [57,58]. Glial activation adds an additional layer of modulation: astrocytes and microglia release cytokines such as interleukin-1β and tumor necrosis factor-α, as well as brain-derived neurotrophic factor, which sustain NMDA receptor phosphorylation and maintain neuronal hyperexcitability [59,60]. Ascending projections from the TNC synapse onto third-order neurons in the ventroposteromedial nucleus of the thalamus, which relay nociceptive signals to cortical regions, including the primary and secondary somatosensory cortices, insula, and anterior cingulate cortex. Maladaptive plasticity within these thalamo-cortical networks contributes to abnormal pain perception, affective amplification, and persistence of pain even after peripheral inputs subside [61]. Collectively, these processes may constitute the neurobiological basis of central sensitization in chronic TMD, particularly chronic myofascial pain manifesting clinically as muscle hyperalgesia and allodynia, temporal summation, and extra-trigeminal pain spread (see this figure). Arrows indicate the direction of nociceptive signal transmission and facilitatory interactions between peripheral and central trigeminal pathways.

Once activated, peripheral nociceptors innervating the TMJ, masticatory muscles, and associated structures undergo sensitization that amplifies and prolongs pain signaling in TMD [38,44]. The afferents of the mandibular branch (V3) of the trigeminal nerve terminate in the TMJ capsule, ligaments, and periarticular tissues [45]. Mechanical overload, ischemia, or inflammation triggers release of excitatory neurotransmitters and neuropeptides such as glutamate, substance P, and calcitonin gene-related peptide (CGRP), promoting neurogenic inflammation and amplifying nociceptive transmission [46,47,48,49]. Ion channels, including transient receptor potential vanilloid 1 (TRPV1) and acid-sensing ion channels, become sensitized in this inflammatory milieu, lowering activation thresholds and sustaining peripheral hyperexcitability [50,51]. Recent evidence indicates that inflammatory mediators play a central role in the pathophysiology of temporomandibular myalgia, with elevated cytokines, chemokines, and prostaglandins contributing to persistent nociceptor sensitization and muscle pain [52]. Within the trigeminal ganglion (TG), interactions between neurons and satellite glial cells further maintain this state through the release of proinflammatory cytokines and chemokines that reinforce nociceptor excitability [32,53,54]. These processes may collectively constitute peripheral sensitization, characterized clinically by local tenderness and a reduced pressure pain threshold (PPT), with pain that is typically evoked or exacerbated by mandibular movement or palpation. Such peripheral events not only initiate local hyperalgesia but also serve as the afferent driver for central sensitization in chronic TMD, which may underlie the development of cutaneous allodynia and widespread pain hypersensitivity. Evidence from clinical studies supports the presence of central sensitization in patients with painful TMD, emphasizing its contribution to symptom persistence and therapeutic challenges [15] (see Figure 2).

## 5. Clinical Manifestations

Patients with chronic TMD present with a heterogeneous constellation of signs and symptoms, including lower sensory thresholds, pain amplification, amplification of the receptive field, and even extracephalic/extratrigeminally areas reflecting involvement of both peripheral and central pain mechanisms [15,40,62].

Chronic myofascial pain may be one of the classic examples of the presence of this central sensitization phenomenon, with persistent pain and the referral pattern of pain beyond the boundaries of the muscle being palpated. This has very important clinical implications, since this referral may even be experienced as dental pain or even otologic complaints, such as ear fullness, tinnitus, or referred otalgia when there is no pathology present in these structures [3,63]. Furthermore, the presence of overlapping pain conditions plays a critical role in the enhancement of central sensitization, maintaining the persistent pain and supporting chronicity. Many patients report coexisting headache symptoms such as headache attributed to TMD, as well as TMD comorbid with migraine or tension-type headache, with migraine having almost twice the prevalence of tension-type headache in these patients, making treatment even more difficult [13,64]. This overlap is highly relevant in clinical practice, where migraine may be misdiagnosed as TMD or, conversely, TMD may be overlooked in patients with migraine. Bruxism is another contributing factor in TMD that may exacerbate, perpetuate, or initiate symptomatology in some individuals [30,64].

In chronic arthralgia and myofascial pain associated with TMD, cutaneous allodynia can occur within both trigeminal and extra-trigeminal regions, indicating that pain amplification extends beyond the initial peripheral site [14,15,20]. Its presence correlates with higher pain intensity, greater functional limitation, and poorer response to conventional therapies, establishing allodynia as a clinical marker of disease severity and prognosis [65]. Hypersensitivity to mechanical and intra-articular electrical stimulation has been demonstrated in individuals with painful temporomandibular joints, further supporting peripheral and central sensitization mechanisms in TMD [14]. Other psychophysical manifestations of central sensitization include reduced PPT and enhanced temporal summation of pain, both consistently observed in TMD cohorts [15].

## 6. Preclinical Mechanistic Evidence

### 6.1. BoNT-A Actions at the Molecular Level

**Figure 3:**
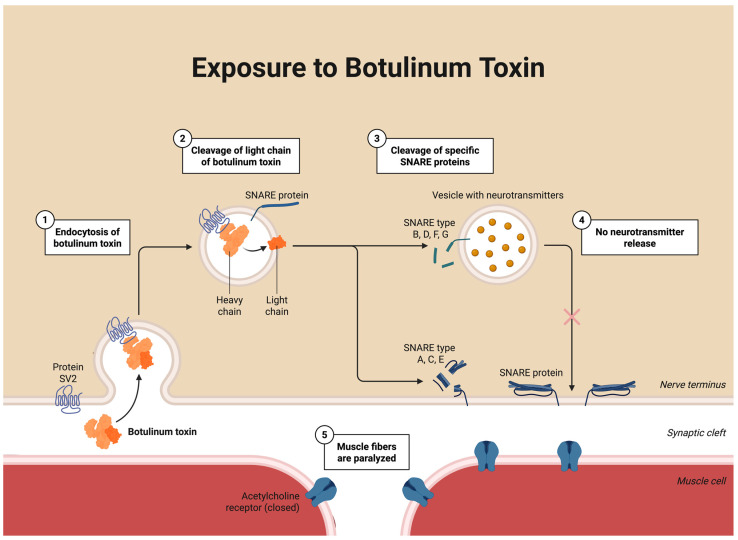
Mechanism of action of botulinum toxin type A at the neuromuscular junction. Botulinum toxin type A (BoNT-A) inhibits neurotransmitter release at the neuromuscular junction by targeting SNARE-mediated vesicle fusion. (1) BoNT-A binds to the presynaptic membrane via synaptic vesicle protein 2 (SV2) and undergoes endocytosis. (2) The toxin’s light chain is released into the cytosol, where it acts as a zinc-dependent endopeptidase. (3) The light chain cleaves the SNARE protein SNAP-25, essential for vesicular docking and acetylcholine release. (4) This blockade prevents exocytosis of acetylcholine into the synaptic cleft, leading to (5) temporary paralysis of the targeted muscle fibers [76]. This figure was created using BioRender.

Understanding how BoNT-A modulates nociceptive processing requires examination of its molecular actions in sensory and motor neurons. The antinociceptive properties of BoNT-A originate from its enzymatic action within presynaptic neurons [66,67]. Structurally, BoNT-A consists of a heavy chain (100 kDa) and a light chain (50 kDa), which function together to mediate neuronal binding, endocytosis, and intracellular proteolysis [67,68]. Following injection, the heavy chain binds to synaptic vesicle glycoprotein 2 on the nerve terminal membrane, initiating endocytosis [67,68]. Endosomal acidification subsequently enables translocation of the light chain into the cytosol [67,68]. Once internalized, the light chain acts as a zinc-dependent endopeptidase that cleaves synaptosomal-associated protein of 25 kDa (SNAP-25), a component of the soluble N-ethylmaleimide-sensitive factor attachment protein receptor (SNARE) complex, which is required for vesicular docking and neurotransmitter release (see Figure 3) [68,69].

These molecular events are not only responsible for muscle relaxation but also form the foundation of BoNT-A’s proposed antinociceptive and anti-allodynic actions. This protelyctic cleavage prevents exocytosis of acetylcholine at the neuromuscular junction, producing muscle relaxation [70], but similar cleavage occurs in sensory neurons, where it suppresses release of excitatory mediators, including glutamate, substance P, and CGRP [71]. Studies in the TG and dorsal root ganglion confirm that BoNT-A reduces depolarization-evoked release of CGRP and substance P [71], supporting a direct sensory modulatory action relevant to TMD pain. In addition, preclinical evidence indicates that BoNT-A can modulate nociceptor sensitivity through noncanonical mechanisms, including reduced trafficking of TRPV1 and purinergic P2X3 receptors to the membrane, and attenuation of glial activation [69,72]. These effects suggest a broader anti-inflammatory and antinociceptive profile beyond SNAP-25 cleavage alone. Together, these cellular effects provide a mechanistic link between BoNT-A and modulation of peripheral nociceptive drive, a prerequisite for reducing central sensitization.

Although such mechanisms offer a coherent biological rationale for targeting both peripheral and central sensitization in TMD, their clinical significance remains unclear [73]. Most mechanistic evidence comes from rodent or in vitro models using toxin concentrations or exposure durations higher than those encountered in humans. The extent of sensory terminals undergoing SNAP-25 cleavage at therapeutic doses, and the duration of truncated SNAP-25 fragments, have not been systematically measured. Moreover, while limited retrograde axonal transport and transcytosis to second-order neurons have been observed in animal models, their role in human central pain modulation is uncertain [74,75].

### 6.2. Peripheral Effects on Nociception and Inflammation

Preclinical evidence demonstrates that BoNT-A reduces neurogenic inflammation and modulates nociceptor excitability [66,76]. In rodent models of orofacial inflammatory pain, peripheral BoNT-A injections decreased plasma extravasation and tissue edema, effects attributed to inhibition of CGRP, and substance P-mediated vascular responses [66,77,78]. In parallel, BoNT-A has been shown to downregulate TRPV1 expression and activity in trigeminal sensory neurons [50,79]. This is of importance, as TRPV1 sensitization contributes to mechanical allodynia and thermal hyperalgesia [80]. Additional data indicate suppression of peripheral inflammatory signaling through reduced activation of mitogen-activated protein kinase and extracellular-signal-regulated kinase (ERK) pathways and decreased expression of interleukin-1β and tumor necrosis factor-α in local tissues [81,82]. Together, these peripheral effects demonstrate that BoNT-A not only interrupts neurotransmitter release but also reduces the excitability of nociceptors and links to the reduction in peripheral sensitization. By diminishing the intensity and frequency of peripheral nociceptive input, these actions may indirectly dampen central excitability and mitigate the development of allodynia [83]. Recent data also suggest an immune component to BoNT-A’s peripheral action [84]. In vivo imaging in female mice showed that BoNT-A shifted meningeal macrophages from a pro- to an anti-inflammatory phenotype after cortical spreading depression. Although derived from migraine models, the trigeminal immune milieu is shared with TMD, supporting a unified framework in which BoNT-A exerts immune-neural modulation across craniofacial pain disorders [85].

Nevertheless, substantial heterogeneity exists among studies. Experimental designs differ in administration route, tissue target, and dosage, with some studies using direct muscle injections, while others deliver BoNT-A perineurally or intra-articularly [66,86,87,88]. These variations affect tissue diffusion, local concentration, and the pattern of neuronal exposure. Furthermore, the endpoints used to infer anti-inflammatory activity, such as plasma extravasation or edema, are indirect and may be influenced by anesthesia, stress, or systemic factors. The mechanistic relevance of TRPV1 modulation is also debated, as it remains unclear whether BoNT-A directly alters channel expression or merely reduces upstream inflammatory signaling [89,90]. Few studies provide time-course data linking TRPV1 downregulation to behavioral improvement, which limits causal interpretation [91,92].

### 6.3. Central Effects of Peripheral BoNT-A Administration

Beyond the periphery, BoNT-A produces measurable downstream effects within central nociceptive circuits, raising the possibility of secondary modulation of central sensitization. Although BoNT-A is administered peripherally, converging evidence from animal studies suggests secondary effects on central nociceptive circuits [35,66]. In models of TMJ inflammation, peripheral BoNT-A injections reduce c-Fos expression in the TNC, indicating diminished activation of second-order nociceptive neurons [35,36,66,93]. Other studies demonstrated suppression of microglial activation and reduced expression of proinflammatory cytokines such as interleukin-1β and TNF-α within the brainstem and spinal dorsal horn [36,94]. Electrophysiological recordings from wide dynamic range neurons revealed attenuated hyperexcitability and decreased responses to noxious stimulation, findings consistent with reduced central sensitization [36,95]. Together, these findings imply that BoNT-A can dampen central nociceptive processing, either through reduced afferent input or limited retrograde signaling along sensory pathways. This reduction in central markers of activation aligns with preclinical evidence in migraine and supports the hypothesis that BoNT-A may indirectly attenuate central allodynia through reduced peripheral drive [36,96].

Interpretation of these data, however, requires caution. The reduction in c-Fos expression, while often interpreted as a central effect, may simply reflect suppression of peripheral nociceptor drive [97,98]. Evidence for retrograde axonal transport of BoNT-A remains indirect, based primarily on immunohistochemical detection of cleaved SNAP-25 fragments in central tissues [34,35,98,99]. Moreover, reports of glial modulation vary in timing, regional specificity, and methodological rigor, making it difficult to distinguish primary central actions from secondary adaptations to reduced peripheral signaling [34,98]. Overall, the weight of evidence indicates that peripheral BoNT-A administration decreases central nociceptive activity in trigeminal circuits, but whether this results from central penetration or downstream modulation of peripheral input remains unresolved. Thus, while preclinical studies consistently demonstrate decreased central excitability following peripheral BoNT-A administration, definitive evidence for direct central action in humans remains elusive and represents a key gap in translation.

### 6.4. Effect on Allodynia

Among preclinical observations, perhaps the most clinically relevant is BoNT-A’s ability to attenuate allodynia, a behavioral marker of central sensitization, in models of pain in trigeminal and temporomandibular disorders [100,101,102]. In inflammatory TMD models, intra-articular or intramuscular BoNT-A reduced mechanical and thermal allodynia [66,82,86]. These changes paralleled decreased activity of TRPV1-positive afferents and reduced release of CGRP and substance P, together with lower expression of sensitization markers, such as phosphorylated ERK and c-Fos in the brainstem, suggesting dampened peripheral drive and downstream central desensitization [66,82,86].

Interpretation of these findings, however, requires caution. Behavioral assays such as von Frey testing are vulnerable to experimenter bias and methodological variability, and blinding or randomization is not consistently reported [102,103]. The duration and dose–response relationships of the anti-allodynic effects also vary substantially across models. Moreover, contralateral improvements may reflect systemic diffusion or autonomic changes rather than genuine central action, and temporal dissociation between molecular markers and behavioral recovery complicates causal inference [66,82,86]. Thus, while preclinical data consistently support an anti-allodynic effect of BoNT-A and position allodynia reversal as a tangible readout of its impact on trigeminal nociceptive processing, the precise mechanisms and their translational relevance to human TMD pain remain to be fully established.

### 6.5. Methodological Considerations and Translational Limitations

Although preclinical studies provide a coherent mechanistic framework for BoNT-A-induced analgesia, several methodological constraints limit translation to clinical contexts. Experimental doses often exceed human-equivalent concentrations, and diffusion from the injection site is rarely quantified, leaving the effective tissue exposure uncertain. Variability in administration routes and timing of assessments further complicates interpretation, as molecular changes are frequently measured hours after injection while behavioral effects emerge days later. Common readouts, such as c-Fos or cytokine levels, are indirect indices of nociceptive modulation, and reproducibility is limited by inconsistent blinding and randomization. Another important limitation concerns model specificity. Many experimental paradigms employ formalin-induced or neuropathic orofacial pain models (e.g., whisker pad or infraorbital nerve stimulation) rather than true representation TMD models as musculoskeletal pains involving the masseter muscle or TMJ. While these approaches capture key trigeminal nociceptive mechanisms, they may not fully reproduce the biomechanical loading, joint microenvironment, chronic myofascial features, and the full complexity and heterogeneity characteristic of human TMD. Consequently, extrapolating these findings to clinical TMD requires caution.

Despite these caveats, the collective evidence supports a multimodal antinociceptive profile of BoNT-A, encompassing inhibition of neuropeptide release, suppression of peripheral inflammation, and attenuation of allodynia that may prevent or revert central sensitization. Refining orofacial models and aligning molecular and behavioral endpoints will be key to improving mechanistic precision and bridging preclinical insights to clinical application.

## 7. Human Clinical Studies

### 7.1. Clinical Efficacy

Interpretation of clinical BoNT-A studies is inherently complicated by the diagnostic diversity encompassed under TMD. More than 30 conditions fall within the DC/TMD taxonomy, each with distinct structural, functional, and psychosocial contributors to pain [10]. Despite this heterogeneity, many painful TMD phenotypes share common pathways of peripheral and central sensitization, providing a mechanistic rationale for applying BoNT-A across these conditions. In human studies, reductions in PPT and quantitative sensory testing (QST) serve as perceptual correlates of peripheral and central sensitization. One of the most methodologically rigorous early randomized controlled trials evaluated BoNT-A in patients with persistent myofascial TMD pain using bilateral masseter and temporalis injections under strict blinding [31]. Although the treatment failed to outperform the placebo on the primary pain endpoint, it produced modest, short-term reductions in pain and small increases in PPT, indicating partial attenuation of mechanical hypersensitivity consistent with reduced allodynia [104,105]. These limited sensory improvements parallel preclinical findings showing that BoNT-A dampens nociceptor excitability and central sensitization [103,105,106], suggesting that its modest clinical effect reflects incomplete engagement of sensitization mechanisms rather than a pure muscle relaxant response.

The largest randomized controlled trial to date, a five-arm dose-ranging study, enrolled 100 women with chronic myofascial TMD pain who received three BoNT-A doses, oral appliance (OA) therapy, or a placebo over 24 weeks [107]. BoNT-A produced significant reductions in pain intensity and increases in PPT versus placebo, reflecting attenuation of mechanical hypersensitivity consistent with reduced allodynia, yet it failed to outperform OA at the study end. Psychosocial scores for pain-related disability, depression, and somatization improved similarly with BoNT-A and OA, underscoring overlapping efficacy. Imaging follow-up revealed dose-dependent declines in masticatory muscle thickness and mandibular bone volume, confirming biological activity but exposing a functional cost at higher doses. Overall, BoNT-A achieved modest analgesia and sensory desensitization relative to placebo but offered no clear clinical advantage over conservative therapy.

In a companion randomized trial comparing three BoNT-A doses with a placebo in 80 women with chronic myofascial TMD pain [108], BoNT-A showed significantly improved pain-free mouth opening and reduced muscle tenderness at 28 and 180 days, with no dose–response gradient. The analgesic effect persisted beyond expected motor recovery, implying mechanisms beyond muscle relaxation. Improvement in mechanical sensitivity mirrored prior PPT increases, consistent with partial reversal of peripheral sensitization. Nonetheless, concurrent electromyography (EMG) studies documented reduced masticatory activity, suggesting that analgesia may occur at the cost of transient functional weakness. Overall, BoNT-A provides a dose-independent sensory benefit but a narrow therapeutic window between pain relief and muscle impairment.

Smaller controlled studies support modest analgesic effects but emphasize methodological variability. In a double-blind randomized trial, 24 patients with myofascial pain were assigned with and without temporomandibular joint disc displacement, to BoNT-A or a placebo in the masseter and temporalis muscles [109]. BoNT-A reduced pain and improved jaw function at 14 and 28 days, accompanied by an approximately 80% transient decline in masseter EMG activity consistent with reversible neuromuscular inhibition. However, the small sample, subjective endpoints, and lack of blinded evaluation limit interpretation. In another study, a comparison of single-session BoNT-A with fascial-manipulation therapy in 30 patients showed significant pain reduction at three months in both groups, with minimal between-group differences and slightly greater improvement after fascial manipulation [110]. However, unbalanced baseline pain levels and the absence of blinding further obscure pharmacologic attribution. Overall, these studies suggest a short-term benefit from BoNT-A but indicate that nonspecific or comparator effects may account for part of the observed improvement.

Arthrogenic and mixed TMD phenotypes demonstrate even greater variability. In a double-blind randomized trial, 15 U of BoNT-A was injected into the lateral pterygoid of 18 patients with painful TMJ clicking, and outcomes were compared with 18 placebo controls [111]. Both groups showed significant pain improvement at three months, but the differences between groups were not significant. BoNT-A modestly increased lateral mandibular movement and transiently reduced click severity, though the low-dose, single-site injection and the small sample size likely limited effect detection. Concomitant NSAID and muscle-relaxant use further confounded the results. These findings suggest that while lateral pterygoid injection may relieve localized discomfort, robust evidence for BoNT-A efficacy in arthrogenic TMD remains absent.

Studies focusing on physiological endpoints have confirmed predictable motor effects. In a crossover RCT, BoNT-A markedly reduced EMG activity and maximal bite force for several months, with full recovery by six to eight months [112]. These findings validate on-target neuromuscular pharmacodynamics but highlight a trade-off between analgesia and muscle function [113].

Two consistent patterns emerge. First, BoNT-A reliably improves mechanical sensitivity (PPT and QST) over global pain ratings, echoing preclinical evidence for modulation of peripheral and central sensitization. Second, although the largest placebo-controlled trial showed efficacy versus placebo [107], the most rigorous earlier placebo-controlled study did not, underscoring that group-level analgesic effects remain modest and sensitive to methodological differences [31]. Taken together, clinical evidence suggests that BoNT-A engages mechanisms related to sensitization modulation but that these effects are small, variable, and constrained by dosage.

### 7.2. Methodological Heterogeneity in Trials

Marked heterogeneity in study design, injection mapping, and outcome selection underlies the inconsistent results across BoNT-A trials in TMD. Doses typically range from 20 to 50 U per masseter and 10 to 30 U per temporalis, yet the number of injection points, per-muscle dose density, and inclusion of additional targets such as the lateral pterygoid or TMJ capsule are rarely standardized. Few studies employ image guidance or validated anatomical grids. One trial [29] used fixed multisite injections under strict blinding and an inert placebo but showed no clear analgesic superiority [31], whereas dose-ranging arms and mechanistic readouts improved interpretability but revealed that analgesic gains were counterbalanced by structural and functional costs [114]. Studies using active comparators such as acupuncture or fascial manipulation complicate attribution of pharmacologic specificity. Investigations emphasizing EMG and bite-force endpoints consistently confirm neuromuscular inhibition without proportionate improvement in pain outcomes.

Outcome heterogeneity further complicates synthesis. Investigators variously report visual analogue scale pain ratings, PPT, jaw function indices, Helkimo scores, EMG, occlusal force, and global-impression scales, but, more importantly, in some, the diagnostic criteria for TMD are not used, and there is no distinction of what type or types of TMD the patient population presented using “TMD” as a diagnosis. Positive findings in one domain often fail to replicate in others. Reporting of randomization, allocation concealment, and power calculations is inconsistent, particularly in smaller trials. These issues underscore the need for standardized primary endpoints and validated multidimensional pain measures in future research. In contrast with migraine research, which benefits from detailed methodological guidelines issued by the International Headache Society (IHS) for acute and preventive treatment trials [115,116], no equivalent standards exist for TMDs. The Diagnostic Criteria for Temporomandibular Disorders (DC/TMD) consortium provides validated diagnostic and psychosocial assessment frameworks but does not specify trial design parameters, such as primary efficacy endpoints, responder thresholds, or control conditions [10]. This methodological gap is complicated by the broad diagnostic spectrum of TMDs. Although more than 30 different disorders fall under the TMD umbrella, BoNT-A is used only for well-selected cases of myogenous TMD, myalgia, and myofascial pain that remain refractory to conservative care. Clear clinical evaluation and precise diagnosis are, therefore, essential, as mixing different TMD subtypes obscures treatment effects and limits interpretability. Development of standardized trial recommendations analogous to IHS guidelines would substantially improve reproducibility and accelerate progress toward evidence-based implementation of BoNT-A therapy in TMD.

### 7.3. Predictor Response

Several studies indicate that baseline sensory phenotype influences responsiveness to BoNT-A [117,118,119]. Patients exhibiting greater mechanical allodynia or evidence of central sensitization appear more likely to achieve meaningful reductions in pain and increases in PPT. Patients with a higher burden of mechanical allodynia or central sensitization, as assessed by QST, appear more likely to benefit from treatment [118,120]. These findings align with mechanistic models in which BoNT-A acts not only by reducing muscle hyperactivity but also by modulating peripheral and central sensitization [87]. In a dose-ranging RCT, a larger relative benefit among participants with lower baseline PPTs was observed, suggesting preferential efficacy in hypersensitive individuals. Smaller cohorts employing QST show similar trends, although these trials were underpowered to test statistical interaction effects. Future prospective studies combining sensory phenotyping with objective functional monitoring are needed to refine candidate selection and optimize dose–response balance.

### 7.4. Safety Considerations

The short-term safety profile of BoNT-A in TMD parallels that seen in other craniofacial applications, with most adverse events being mild, local, and reversible. Common effects include chewing weakness, transient facial asymmetry, and dysphagia attributable to toxin diffusion into adjacent muscles [99]. Electromyography and occlusal force studies corroborate sustained but reversible reductions in muscle activity following injection [100,101]. Localized muscle atrophy has also been reported following BoNT-A administration in masticatory muscles, consistent with reduced muscle activation and unloading. Experimental and imaging studies suggest that prolonged reduction in masticatory muscle activity may alter mandibular loading and temporomandibular joint biomechanics, with potential downstream effects on alveolar and mandibular bone density. One study documented dose-related decreases in muscle thickness and reductions in mandibular condylar and coronoid bone volume [21]. Preclinical data indicate that prolonged unloading can induce local bone remodeling and reduced mineral density [121,122,123]. Although the long-term clinical relevance and reversibility of these structural changes remain incompletely understood, they warrant careful consideration, particularly with repeated injections or higher cumulative doses. Accordingly, restrained dosing strategies, extended reinjection intervals, and periodic functional or imaging surveillance may be prudent for patients receiving repeated BoNT-A treatments. Future clinical trials should incorporate objective monitoring of muscle function and craniofacial bone integrity to better define the long-term safety profile of BoNT-A in TMD. Hence, while BoNT-A appears safe and reversible, optimizing dosing to balance anti-sensitization efficacy with preservation of masticatory function remains a critical clinical and mechanistic challenge.

## 8. Conclusions

Although multiple systematic and umbrella reviews have evaluated the clinical efficacy of BoNT-A for TMDs and reported overall moderate evidence with substantial heterogeneity, the present review focuses on integrating experimental, translational, and clinical data to elucidate the mechanistic basis underlying heterogeneous clinical responses and to inform mechanism-driven patient stratification and trial design. The use of BoNT-A in TMD is not FDA-approved, and it is not the first line of management; therefore, it is only reserved for refractory cases [124]. However, preclinical and clinical evidence suggest that BoNT-A may modulate nociceptive processing in TMD by reducing peripheral excitability and central sensitization, with possible attenuation of mechanical allodynia. These effects may extend beyond muscle relaxation, though clinical efficacy remains modest and variable. Clinical trials remain heterogeneous, with mixed efficacy but signals of benefit in sensitized subgroups, emphasizing the need for stratified, mechanism-based treatment approaches. Future research should employ standardized sensory phenotyping, optimized dosing, and objective monitoring of muscle and bone safety to identify patients most likely to benefit from BoNT-A. Within multimodal management, BoNT-A could be considered as a potential adjunctive, mechanism-based therapy. Development of consensus methodological guidelines, similar to those in migraine research, will be essential to improve reproducibility and accelerate clinical translation.

## Data Availability

No new data were created or analyzed in this study.

## Abbreviations

The following abbreviations were used in this manuscript:

BoNT-A	Botulinum toxin type A
CGRP	Calcitonin gene-related peptide
EMG	Electromyography
ERK	Extracellular-signal-regulated kinase
IHS	International Headache Society
NMDA	N-methyl-D-aspartate
OA	Oral appliance
PPT	Pressure pain threshold
QST	Quantitative sensory testing
RCT	Randomized clinical trial
TMDs	Temporomandibular disorders
TMJ	Temporomandibular joint
TG	Trigeminal ganglion
TNC	Trigeminal nucleus caudalis
TRPV1	Transient receptor potential vanilloid-1
VPM	ventroposteromedial nucleus
